# Novel loop-mediated isothermal amplification (LAMP) assay with a universal QProbe can detect SNPs determining races in plant pathogenic fungi

**DOI:** 10.1038/s41598-017-04084-y

**Published:** 2017-06-26

**Authors:** Yu Ayukawa, Saeri Hanyuda, Naoko Fujita, Ken Komatsu, Tsutomu Arie

**Affiliations:** 1 United Graduate School of Agricultural Science, Tokyo University of Agriculture and Technology (TUAT), Fuchu, Tokyo, 183-8509 Japan; 2 Laboratory of Plant Pathology, Graduate School of Agriculture, Tokyo University of Agriculture and Technology (TUAT), Fuchu, Tokyo, 183-8509 Japan

## Abstract

Tomato wilt pathogen *Fusarium oxysporum* f. sp. *lycopersici* (*Fol*) is grouped into three races based on their pathogenicity to different host cultivars. Rapid detection and discrimination of *Fol* races in field soils is important to prevent tomato wilt disease. Although five types of point mutations in *secreted in xylem 3* (*SIX3*) gene, which are characteristic of race 3, have been reported as a molecular marker for the race, detection of these point mutations is laborious. The aim of this study is to develop a rapid and accurate method for the detection of point mutations in *SIX3* of *Fol*. Loop-mediated isothermal amplification (LAMP) of *SIX3* gene with the universal QProbe as well as two joint DNAs followed by annealing curve analysis allowed us to specifically detect *Fol* and discriminate race 3 among other races in about one hour. Our developed method is applicable for detection of races of other plant pathogenic fungi as well as their pesticide-resistant mutants that arise through point mutations in a particular gene.

## Introduction

*Fusarium oxysporum* f. sp. *lycopersici* (*Fol*) is a causal agent of tomato wilt. *Fol* invades tomato roots, colonizes vessels and finally kills the host plants. *Fol* has three races, which are classified based on virulence on tomato cultivars carrying different resistant genes (*Immunity*: *I*)^1^. Cultural control of *Fol* race 3 is laborious because there is no commercial tomato cultivar that is resistant against the race. Tomato rootstock carrying I, I2 and I3 or soil disinfection are needed to control outbreak of race 3. Since its first occurrence in Australia in 1978, *Fol* race 3 has been reported from other areas including the United States, Mexico and Brazil^2–8^. In Japan, *Fol* race 3 was firstly isolated in Fukuoka prefecture in 1997^9^, followed by its occurrences in Hokkaido, Kumamoto, Kochi, Aomori and other prefectures^10, 11^. Under these situations, stable tomato production requires earlier detection and discrimination of *Fol* races for the selection of appropriate resistant cultivars and rootstocks.

*Fol* has race-specific *avirulence* (*AVR*) genes. Recent studies have identified some of the small secreted proteins of *Fol* in tomato xylem (secreted in xylem: SIX) as products of *AVR* genes^12–14^. As SIX4, SIX3 and SIX1 were recognized by I/I-1, I2 and I3, each SIX protein was designated as AVR1, AVR2 and AVR3, respectively^12–14^. While *Fol* race 1, which harbors *SIX4* (*AVR1*), cannot infect tomato cultivars possessing *I* or *I-1*(I/I-1) due to the recognition of SIX4 by I or I-1, races 2 and 3 can break this resistance by escaping recognition by I/I-1 based on the mutation in their *SIX4* gene, such as deletions and transposon insertions in its open reading frame^11, 13, 15^. *Fol* races 1 and 2 cannot exhibit virulence on *I*/*I-1 I2* tomato cultivars because I2 recognizes SIX3 (AVR2) expressed by both races. However, *Fol* race 3 can infect *I/I-1 I2*-harboring cultivars since a single point mutation (G121A, G134A or G137C) in its *SIX3* gene evades I2-mediated resistance^14^. In addition to these three reported mutation types, two new types of a single point mutation (T122A or C146T) in the *SIX3* have been recently found from *Fol* race 3 isolates in Japan (Akai *et al*., in preparation). Collectively, *AVR* genes of *Fol* have diverse types of mutations that cause race differentiation. Therefore, identifying mutation types of *AVR* genes of *Fol* is important for determination of its races.

PCR- or real-time PCR-mediated *Fol* race distinction method has been developed by targeting their *AVR* genes^16, 17^. This allowed discrimination between *Fol* race 1 and other races by detection of *SIX4*, as well as identification of *Fol* race 3 by primers that can detect either of the three types of single nucleotide substitutions (G121A, G134A or G137C) in *SIX3*. To improve rapidity and sensitivity of *Fol* race distinction, we have recently developed a loop-mediated isothermal amplification (LAMP) method targeting *SIX4* unique to race 1^18^. However, LAMP has never been applied to the discrimination of *Fol* race 3 from other races, which needs detection of point mutations in *SIX3*.

In order to detect single nucleotide polymorphisms (SNPs) by LAMP, one approach is to use an inner primer possessing a specific 3′- or 5′-terminal nucleotide that anneals to the corresponding SNP site, which allows specific amplification of a target gene containing SNPs^19–25^. However, this method is costly, because it needs a different inner primer for detection of different types of mutations of the target gene.

Using quenching probe (QProbe) could be another approach for detection of SNPs by LAMP. QProbe is a single-stranded nucleotide, the 5′ end of which is a cytosine labeled with a fluorophore such as BODIPY or fluorescein^26, 27^. When QProbe hybridizes with its target nucleotide sequence, the fluorescence is quenched by electron transfer between the fluorophore and a guanine residue in the target. The fluorescence of QProbe is recovered upon its dissociation from the target. These properties of QProbe allow it to be used for SNP typing in combination with real-time PCR and subsequent melting curve analysis^26^. In other words, one can determine whether there are SNPs or not by analyzing melting temperature of QProbe, which will be lower with a target sequence containing a mismatch with the QProbe, compared to a perfect match sequence. However, detection of different SNPs in a relatively distant region of the same gene requires additional synthesis of the fluorescent QProbe, which is costly and time-consuming. To alleviate this problem, universal QProbe system has been developed^28^. This method uses a universal QProbe combined with a joint DNA, instead of a single, target region-specific QProbe. Nucleotide sequences of a universal QProbe can be fixed because a joint DNA is designed to contain two combined sequences complementary to both a target region and a universal QProbe. Therefore, this method is less time-consuming and more cost-effective.

In this study, we tried to apply the universal QProbe system to LAMP in order to detect five types point mutations of *SIX3* in *Fol* race 3. This is the first report of the successful combination of LAMP and the universal QProbe, which achieved rapid discrimination of *Fol* race 3 from other two races.

## Results

### Design of LAMP primer set and joint DNAs, and optimization of LAMP conditions

**Figure 1:**
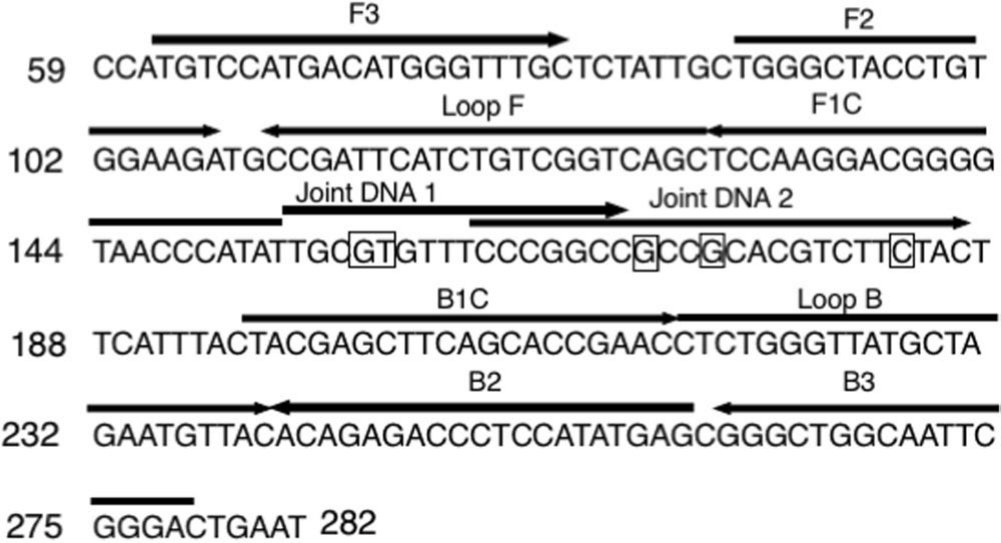
Design of LAMP primers and joint DNAs Partial *SIX3* nucleotide sequences (positions 59 to 282) used for designing LAMP primers and joint DNAs. Positions of the designed primers and joint DNAs are indicated by arrows. Point mutation sites are shown by an open box.

**Figure 2:**
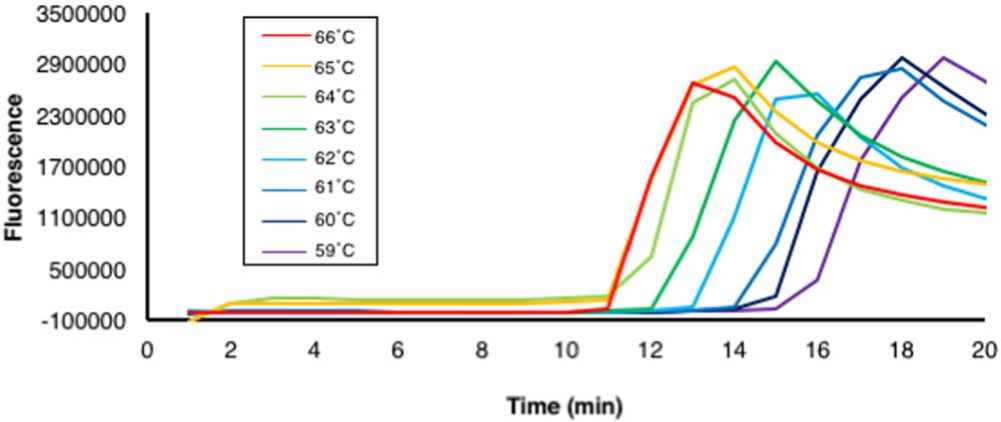
Optimization of LAMP reaction temperature with the designed *SIX3* primer sets. LAMP was performed with DNA of *Fol* race 1 at different temperatures from 59 to 66 °C for 20 min. Similar results were obtained in two independent experiments.

To distinguish *Fol* race 3 from other races by LAMP combined with the universal QProbe system, a primer set for a basic LAMP method was designed based on the *SIX3* nucleotide sequence (GenBank accession number: AM234063.1) (Fig. 1, Table 1). Joint DNA1 and 2 were designed to hybridize to nt 113–129 and 127–149, respectively, with respect to a translation initiation codon of *SIX3*. Before determination of melting temperatures of the two joint DNAs combined with the universal QProbe, we examined optimal reaction temperature for LAMP by the *SIX3* primer set without the QProbe and joint DNAs. When LAMP was performed with genomic DNA (gDNA) (30 ng) of *Fol* race 1 (MAFF 103036) in the range of 59 to 66 °C, the reaction at 65 or 66 °C showed the most rapid increase in the fluorescence intensity in about 10 min (Fig. 2). Thus, all subsequent LAMP assays were performed at 66 °C.

**Table 1:** Melting temperatures of Joint DNAs.

	Strains	Origin	SIX3 mutation types	Annealing temperature (arithmetic mean °C ± standard deviation)	
				Joint DNA1	Joint DNA2
*Fusarium oxysporum* f. sp. *lycopersici* race 1	MAFF 103036	Japan	None	59.3 ± 0.2	71.6 ± 0.5
	MAFF 305121	Japan	None	61.1 ± 1.2	72.4 ± 0.4
race 2	JCM 12575	Japan	None	60.7 ± 1.0	71.7 ± 0.3
	4287	Spain	None	60.4 ± 2.2	73.1 ± 0.3
race 3	Chz1-A	Japan	G121A	53.2* ± 0.4	72.3 ± 0.9
	KoChi-1	Japan	G121A	53.7* ± 1.1	70.2 ± 0.0
	FolyA007	Japan	T122A	54.6* ± 1.0	71.9 ± 0.1
	F240	USA	G134A	60.1 ± 1.0	65.5* ± 1.8
	40-1	Japan	C146T	61.6 ± 2.0	39.3* ± 0.5
	KC0071	Japan	C146T	60.2 ± 0.0	40.3* ± 0.5
*F. oxysporum* f. sp. *batatas*	MAFF 103070	Japan	—	—	—
*F. oxysporum* f. sp. *conglutinans*	Cong:1-1	Japan	—	—	—
*F. oxysporum* f. sp. *nicotianae*	ATCC 15645	Greece	—	—	—
*F. oxysporum* f. sp. *radicis-lycopersici*	MAFF 103044	Japan	—	—	—
*F. oxysporum*	Fo304	Japan	—	—	—
*F. sacchari*	FGSC 7611	USA	—	—	—
*Alternaria solani*	AS2	Japan	—	—	—
*Verticillium dahliae*	910312a-3	Japan	—	—	—
plasmid DNA	pSIX3 G137C		G137C	59.7 ± 0.1	63.8* ± 0.5

+, Detection −, no detection *, relatively low melting temperature of each joint DNA.

### Detection of a point mutation in *SIX3* by LAMP reaction with the universal QProbe system

**Figure 3:**
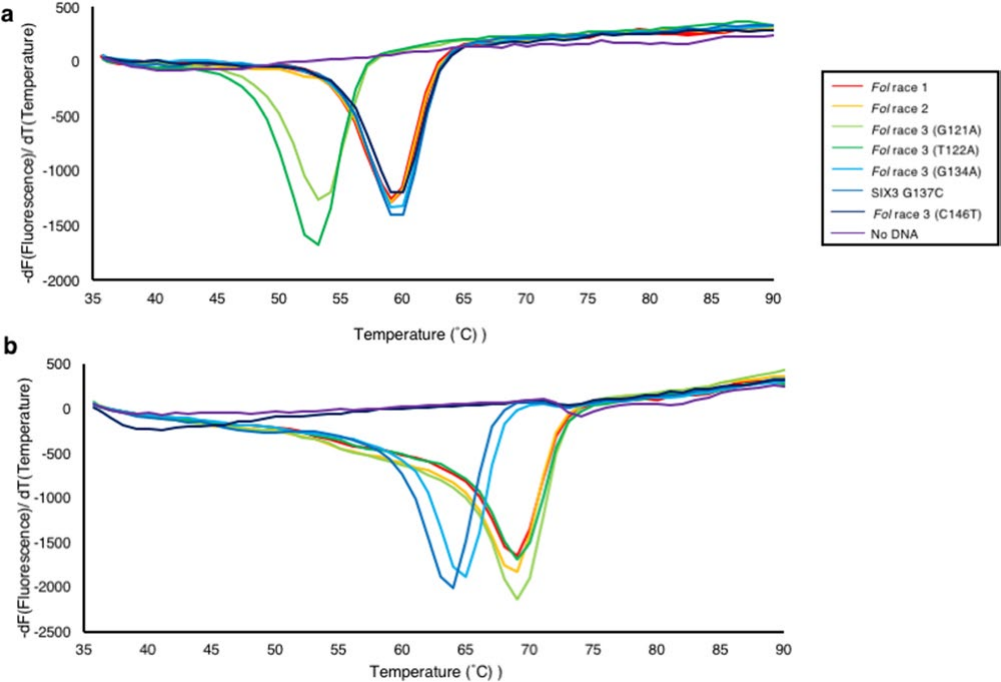
Melting temperature of joint DNAs combined with the universal QProbe. Melting curve analysis with Joint DNA 1 (**a**) or Joint DNA 2 (**b**) was performed from 35 to 90 °C following 60 min of LAMP reactions using genomic DNAs of *Fol* isolates of different races as a template. Similar results were obtained in two independent experiments.

To test the specificity of the designed primer set and joint DNAs, we performed LAMP reactions with the QProbe and Joint DNA 1 or 2 using gDNA (30 ng) of two isolates of *Fol* race 1, two of *Fol* race 2, six of *Fol* race 3 harboring a single point mutation (two for G121A, one T122A, one G134A or two T146A) in *SIX3*, four of other f. spp., and one of nonpathogenic *Fusarium oxysporum*, and two of other fungal species. We also tested plasmid DNA (3 ng) containing a *SIX3* sequence with another type of single point mutation (G137C), which has not been found in Japan at present. Melting curve analysis following 60 min of LAMP reaction with Joint DNA 1 showed that the negative derivative of fluorescence over temperature produced a single peak at about 60 °C when gDNA of *Fol* race 1, race 2, race 3 (G134A or C146T) or the plasmid DNA (G137C) was used as a template (Fig. 3a, Table 2). On the other hand, a single peak at about 53 °C was observed when gDNA of *Fol* race 3 with a mutation type G121A or T122A was used as a template (Fig. 3a, Table 2). Melting curve analysis following LAMP reaction with Joint DNA 2 produced a single peak at about 70 °C when using gDNA of *Fol* race 1, race 2, or race 3 with a mutation type G121A or T122A, and a single peak at about 65 °C when using *Fol* race 3 with a mutation type G134A or plasmid DNA with a G137C mutation in *SIX3* (Fig. 3b, Table 2). In this case with Joint DNA 2, a weak single peak at about 40 °C was produced when gDNA of *Fol* race 3 with a mutation type C146T was used as a template. It should be noted that, because we successfully observed a clear peak at 60 °C with Joint DNA 1 when C146T was used as a template, this weak peak observed with Joint DNA 2 might be due to weak annealing of the amplified product of *SIX3* of *Fol* race 3 (C146T) with Joint DNA 2, but not due to failure of amplification. In contrast, no peak was observed by melting curve analysis with Joint DNA 1 or 2 when gDNA of the other f. spp. or non-pathogenic isolates of *F. oxysporum*, that of other fungal species, or water was used as a template, suggesting that the *SIX3* primers are highly specific and do not generate any amplification products when LAMP reaction does not contain DNA of *Fol*. These results indicated that our LAMP method with the universal QProbe system could not only distinguish between *Fol* race 3 and other races and species, but also specifically identify *Fol* race 3 harboring C146T in *SIX3*.

**Table 2:** Primers and joint DNAs used in this study.

Primer	Sequences 5′-3′	Use	Reference
SIX3-F3	TGTCCATGACATGGGTTTGC	*SIX3* detection	This study
SIX3-B3	CAGTCCCGAATTGCCAGC		This study
SIX3-FIP	TATGGGTTACCCCGTCCTTGGA-TGGGCTACCTGTGGAAGA		This study
SIX3-BIP	ACGAGCTTCAGCACCGAACC-CGCTCATATGGAGGGTCTCT		This study
SIX3-LF	GCTGACCGACAGATGAATCGG		This study
SIX3-LB	TCTGGGTTATGCTAGAATGTTACAC		This study
Joint DNA 1	TGCGTGTTTCCCGGCC - UQprobe*		This study
Joint DNA 2	CCCGGCCGCCGCACGTCTTCTAC - UQprobe*		This study
SIX3 F	TATATTACCGACCATCTTGCCTAAACATTTACCA	*SIX3* mutagenesis	This study
SIX3 R	GCCAAGGGGAACTGCCACAG		This study
G137CF	TGCGTGTTTCCCGGCCGCCCCACGTCTTCTACTTCATTTACTA		This study
G137CR	AAATGAAGTAGAAGACGTGGGGCGGCCGGGAAACACGCAAT		This study
SIX4 F	ACTCGTTGTTATTGCTTCGG	*SIX4* detection	Inami *et al*.^11^
SIX4 R	CGGAGTGAAGAAGAAGCTAA		Inami *et al*.^11^
SIX4-F3	TCCAGTTGGAGCAAGTTGG		Ayukawa *et al*.^18^
SIX4-B3	TGCCCGTCTCTGCGATAG		Ayukawa *et al*.^18^
SIX4-FIP	GCCTCCTTGTCATCTACCGCATTTGGGGTGATAGAGAGCCTG		Ayukawa *et al*.^18^
SIX4-BIP	ATGGCAAAGTTGTCACGCGTGTTGGGCCTTGAGTCGAATG		Ayukawa *et al*.^18^
SIX4-LF	GACGGAGAGCAAGTAGCGT		Ayukawa *et al*.^18^
SIX4-LB	CAGGAAAGCCAGGAATAGGACGA		Ayukawa *et al*.^18^

*3′ ends of joint DNAs consist of UQprobe-G complementary sequences

### Comparative sensitivity test of LAMP and PCR

**Figure 4:**
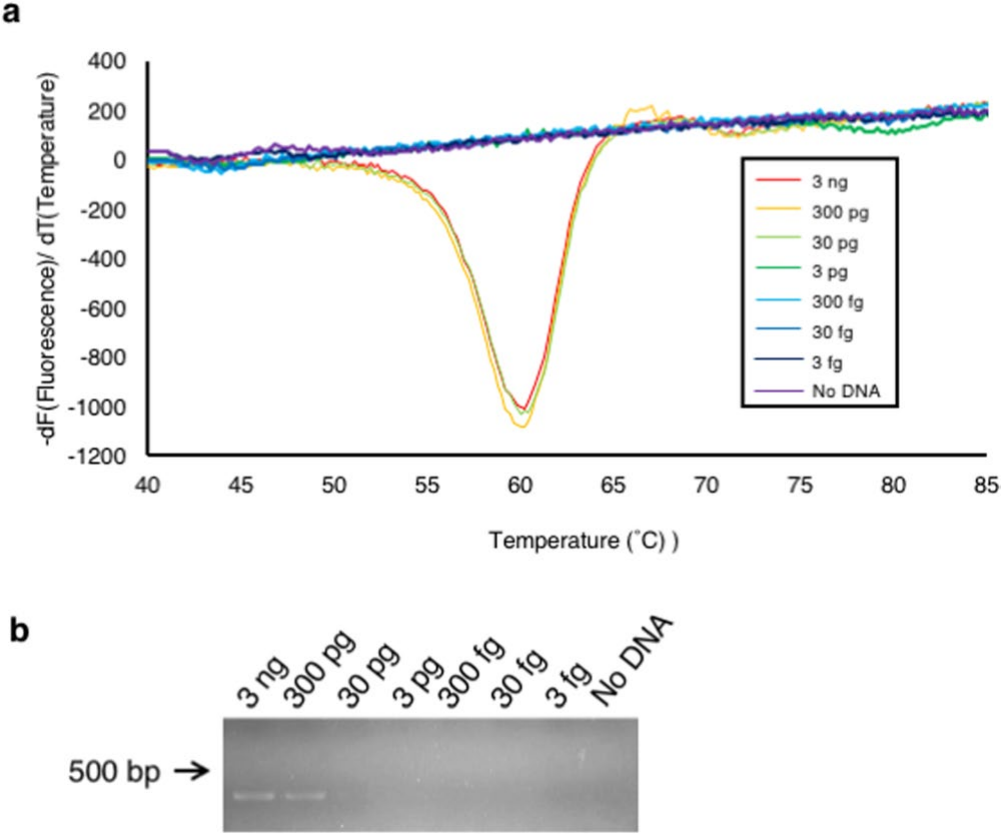
Comparison of relative sensitivity of LAMP with the universal QProbe and conventional PCR (**a**) Melting curve analysis following LAMP reaction with serial tenfold dilutions of genomic DNA of *Fol* race 1 (3 ng to 3 fg) using Joint DNA 1. (**b**) Agarose gel electrophoresis of PCR products amplified from the same dilutions of genomic DNA with SIX3-F3 and SIX3-B3 primers.

To compare the sensitivity of our LAMP method with the conventional PCR, LAMP reaction with the QProbe and the Joint DNA 1, as well as the conventional PCR using outer primers of the LAMP primer set, was performed using a 10-fold dilution series of *Fol* race 1 gDNA, from 3 ng to 3 fg, as a template. In the melting curve analysis following LAMP reaction, the derivative of fluorescence over temperature produced a peak at about 60 °C when 3 ng to 30 pg of DNA was used, while it does not produce any peaks when 3 pg or less DNA was used (Fig. 4a). However, with a template of 3 ng to 300 pg of gDNA, the conventional PCR with the outer primers of LAMP produced amplification products of *SIX3* (Fig. 4b). These results showed that the sensitivity of our LAMP method with the QProbe and the joint DNA was 10-fold higher than that of PCR.

### Detection of *Fol* race 3 from artificially infested soil

**Figure 5:**
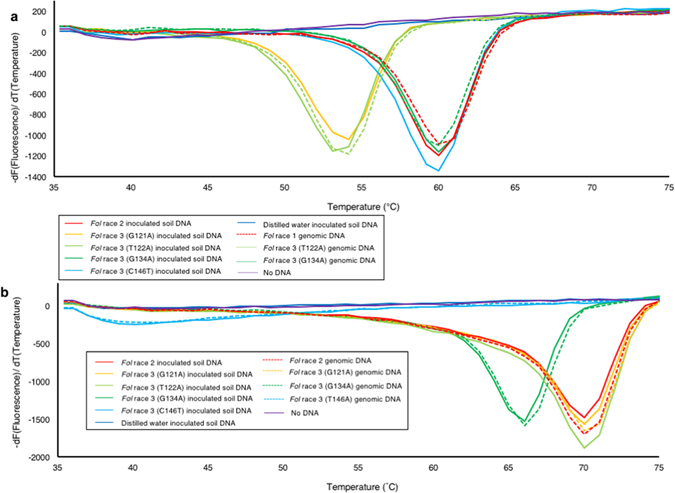
Typing of *Fol* races in artificially infested soil by LAMP using the universal QProbe with joint DNAs. Melting curve analysis of LAMP products using the QProbe combined with Joint DNA 1 (**a**) and Joint DNA 2 (**b**). Genomic DNAs of *Fol* or artificial infested soil DNAs by *Fol* were used as a template. Similar results were obtained in two independent experiments.

To examine the utility of our LAMP method for typing of *Fol* races in the soil, DNA extracted from artificially *Fol*-infested soil was used for LAMP reaction with the QProbe and the joint DNA. Melting temperature of Joint DNA 1 combined with the QProbe was about 54 °C when DNA from soil infested with *Fol* race 3 (with a mutation type G121A or T122A) was used as a template for LAMP reaction, which was lower than that observed when DNA from soil infested with race 3 isolates with other mutation types (G134A and C146T) was used (about 60 °C) (Fig. 5a). On the other hand, melting temperature of Joint DNA 2 combined with the QProbe was about 66 and 40 °C when using DNA extracted from soil infested with *Fol* race 3 of a mutation type G134A and T146A, respectively (Fig. 5b). Accordingly, our method could detect *Fol* race 3 and distinguish it from other races even when using the artificially infested soil DNA.

### Detection of *Fol* race 3 from naturally infested soil

We compared the reliability of our method with that of conventional PCR using soil DNA from two fields (Soil DNAs 1 and 2), which have a history of tomato wilt occurrence by *Fol* race 3 (KoChi-1; G121A in *SIX3*) in 2012 and 2013 (Table 3). Melting temperatures between the LAMP products of both Soil DNA 1 and 2 and the Joint DNA 1 and 2 with the QProbe were in good agreement with that obtained when fungal gDNA of KoChi-1 was used (Table 3). These results suggested that Soil 1 and 2 are infested with *Fol* race 3 isolates carrying G121A or T122A. In contrast, a peak of the derivative of fluorescence over melting temperature was not produced when Soil DNA 3 and 4, which were extracted from rhizosphere soil of healthy tomato plants, were used as a template (Table 3). On the other hand, the conventional PCR with *SIX3* outer primers did not generate amplification from any of the four soil DNAs (Table 3, Supplementary Fig. S1), suggesting that LAMP is more sensitive than the conventional PCR when soil DNA was used as a template.

**Table 3:** Comparison of molecular detection for *Fol* race 3 from field soil of Kochi prefecture.

Sample	Sampled date	Symptom	Location	LAMP with the universal QProbe		LAMP	PCR	
				Melting temperature (arithmetic mean °C ± standard deviation)				
				Joint DNA 1	Joint DNA 2	SIX4	SIX3	SIX4
DNA of KoChi-1	—	—	—	53.5 ± 0.2	70.1 ± 0.3	+	+	+
Soil DNA 1	2012.5	Yes	N33°31′92″ E133°21′97.1″	53.4 ± 0.4	70.0 ± 0.1	+	—	+
Soil DNA 2	2013.5	Yes	N33°31′50.28″ E133°22.01′66″	53.8 ± 0.2	69.7 ± 0.4	+	—	—
Soil DNA 3	2012.11	No	N33°31′92″ E133°21′97.1″	—	—	—	—	—
Soil DNA 4	2014.11	No	N33°31′50.28″ E133°22.01′66″	—	—	+a	—	—

+, detection −, no detection a, LAMP products were obtained only once in three replicates.

To further characterize the race 3 isolates detected from the Soil 1 and 2, we tried to detect their *SIX4* gene, because we have previously reported that the *SIX4* gene of the original KoChi-1 isolate is disrupted by a transposon insertion^11^. For this purpose, we employed the conventional LAMP developed in the previous study^18^, as well as the conventional PCR. LAMP products with the *SIX4* primer set were obtained in three independent reactions when Soil DNA 1 and DNA 2 were used as a template (Table 3, Supplementary Fig. S1). By contrast, a PCR product of *SIX4* was only obtained from the Soil DNA 1 (Table 3, Supplementary Fig. S1), whose molecular size (about 1500 bp) was almost the same as a PCR product of the *SIX4* of the original KoChi-1 isolate, which was inserted with a *Hormin* transposon (Supplementary Fig. S1). These results by the conventional LAMP and PCR suggested the existence of race 3 isolates KoChi-1 in the tested soil, which supported the results of LAMP using the universal QProbe system.

## Discussion

We developed a novel LAMP method with the universal QProbe for detecting five different types of point mutations in *SIX3*, which is characteristic of race 3 of the tomato wilt fungus (*Fol*), and could distinguish race 3 from races 1 and 2. Our method is more accurate and rapid for detecting *Fol* race 3 than other methods based on conventional PCR. PCR targeting polygalacturonase genes or rDNA intergenic spacer region did not completely discriminate *Fol* race 3 from other races or f. spp.^17, 29^, because phylogeny based on these genes does not strictly correlate with races of *Fol*. Although Lievens *et al*.^16^ have developed a PCR-mediated method for detecting *Fol* race 3 based on point mutations in *SIX3*, a race-determining gene, it can only detect three (G121A, G134A and G137C) out of the five types of point mutations found in race 3. Besides, this PCR-mediated detection takes two to three hours. Our LAMP method followed by a melting curve analysis presented that amplification by LAMP is specific for *Fol* and that the melting temperature of QProbe allows discrimination between race 3 and other races in about one hour. However, we cannot exclude the possibility that our method, and also PCR, may accidentally detect *SIX3* homologous gene in another f. sp. Indeed, a *SIX3* homologous gene has been identified from f. sp. *cepae* isolated from onion^30^. Although this *SIX3* homologous gene had a high degree of sequence homology with *SIX3* of *Fol*, there were a few different nucleotides in regions where our joint DNAs can anneal; the *SIX3* sequence of f. sp. *cepae* contains three nucleotide residues different from that of *Fol* at nts. 121, 138 and 139 (G, C and A in *Fol* and C, G and C in f. sp. *cepae*). It is therefore highly likely that we can differentiate f. sp. *cepae* from *Fol* by our melting curve analysis, which will yield lower melting temperature with gDNA of f. sp. *cepae* than *Fol*.

Furthermore, the LAMP method developed in this study is more efficient and versatile to detect several types of point mutations than conventional LAMP. Conventional LAMP with the special inner primers, 5′- or 3′-end of which corresponds to a point mutation site, is able to specifically amplify target nucleotide sequences containing the mutation^19–25^. However, in this method, one needs to design each specific inner primer for different types of nucleotide substitutions. Moreover, this method based on a specific inner primer cannot be combined with loop primers, which accelerate LAMP reaction, due to the unspecific amplification of target genes^22, 24, 25^. By contrast, the method presented in this study exploits loop primers and two joint DNAs, instead of primers specific to each mutation, which can detect five types of point mutations with higher sensitivity. However, it should be also noted that, when we used DNA of *Fol* race 3 (*SIX3* mutation type: C146T) for LAMP reaction with Joint DNA 2, we observed small peak at about 40 °C. Our Qprobe-mediated melting curve analysis is preceded by DNA amplification with conventional LAMP, which used the same primer set regardless of the Joint DNA used for the melting curve analysis. Because a single peak of a derivative of fluorescence was observed when using DNA of *Fol* race 3 (C146T) and Joint DNA 1, it is certain that the primer set used for LAMP works and amplification of DNA is successful. This will also be the case with analysis using Joint DNA 2. Considering that a peak height of the derivative depends on amount of joint DNA combined with amplification product, we estimate that, although amplification step is successful, Joint DNA 2 is not easy to bind to the amplification products containing C146T mutation. In principle, our method can detect single point mutations introduced in a partial *SIX3* sequence covered by two joint DNAs (nucleotide positions 118 to 149). In addition, our method probably allows simultaneous detection of genomic DNAs of *Fol* race 3 isolates and those of *Fol* isolates of other races in a single tube based on difference in melting temperature; two peaks corresponding to race 3 and other races will be observed. Indeed, melting curve analysis with QProbe has been applied for homozygous or heterozygous SNP genotyping^28^. Accordingly, LAMP method with the universal QProbe can replace conventional PCR method to detect *Fol* race 3.

*Fol* race 3 isolates have been reported to have various types of mutations in their *SIX3* gene. Besides the five types of single point mutation subjected in this study, two new mutation types of *SIX3* have been recently found in *Fol* race 3 isolates from Australia^31^. One is deletion of a threonine residue at 50 of *SIX3*. Our method is likely to detect this mutation because an amino acid residue at 50 (nucleotide positions 148 to 150) is included in a region covered by joint DNA 2 (nucleotide positions 127 to 149). Another mutation is a complete loss of *SIX3* ^31^. This may not be detected by our method, but combination of *SIX3* primers and other LAMP primer sets would enable us to detect this mutation. One of the most promising candidates of these primer sets is a combination of *SIX5* and *SIX1*. *SIX5* has been discovered only from *Fol* and f. sp. *cepae* while *SIX1* from other f. sp. except f. sp. *cepae* ^30, 32^. Although these genes are useful to discriminate *Fol* from other ff. spp., we should repeatedly verify the usefulness of our *SIX3*-based system and modify it to cover as many race 3 isolates as possible because *SIX3* mutation plays a key role in the occurrence of race 3 in *Fol*.

Now that we have developed discrimination method of *Fol* race 3 isolates from those of other races, all races of *Fol* isolates can be identified by LAMP. Our present method is unable to distinguish race 1 and race 2, but we have previously developed conventional LAMP to discriminate *Fol* race 1 from other races by detection of *SIX4*, which is unique to *Fol* race 1^18^. Combination of both our LAMP methods can identify almost all *Fol* races. However, it should be noted that *Fol* race 2 isolate Chiba-5 harboring transposon-inserted *SIX4* is indistinguishable by our LAMP methods^15^. We have discriminated transposon-inserted *SIX4* of *Fol* race 3 isolate KoChi-1 from *SIX4* of *Fol* race 1 using LAMP primers targeting the transposon-insertion site^18^. However, the transposon-insertion site of Chiba-5 is different from that of KoChi-1^15^. Chiba-5 could be also distinguished from *Fol* race 1 by designing a new set of LAMP primers that binds to the transposon-insertion site in *SIX4* of Chiba-5.

Our LAMP assay is applicable for diagnosis of tomato wilt using crude DNA from soil. The LAMP with the universal QProbe enabled us to detect *Fol* race 3 from Soil DNA1 and 2 with a history of tomato wilt but not from rhizosphere soils of healthy tomato plants. In contrast, PCR with *SIX3* primers showed no amplification from all soil DNAs tested including DNA1 and 2, which demonstrated superiority of our method. As point mutations in an avirulence gene change races of the plant pathogen, those in a gene targeted by fungicides confer resistance to the drug in several fungal species^33, 34^. LAMP with the universal QProbe presented in this study could lead to the development of novel SNP discrimination methods for understanding plant pathogens in the field.

## Materials and Methods

### Fungal strains and culture conditions

*Fusarium oxysporum* f. sp. *lycopersici* (*Fol*) and other fungal species used in this study are listed in Table 1. These isolates were grown on potato sucrose agar (PSA) medium at 25 °C under dark conditions. For DNA isolation and preparation of *Fol*-infested soil, all isolates were grown on potato sucrose broth (PSB) at room temperature with shaking at 120 spm.

### Sampling soil from field

Soils infested with *Fol* race 3 were sampled from two fields (latitude, N33°31′92″; longitude, E133°21′97.1″; altitude 19 m and latitude, N33°31′50.28″; longitude, E133°22.01′66″; altitude 22 m) in Hidaka, Kochi in May in 2012 or 2013. Rhizosphere soils of healthy tomato plants were sampled from the same fields in January in 2013 or 2014. *Fol* race 2 resistant cultivar Momotaro Fight (Takii Seed, Kyoto, Japan) had been grown in the fields since 2011. All soils were stored at 4 °C before DNA isolation.

### Preparation of artificial infested soil by *Fol*

To prepare soil artificially infested with *Fol*, soil (10 g) (Nippi engei baido No. 1, Nihon Hiryo, Tokyo, Japan) autoclaved for 20 min twice with the interval of 1 h were inoculated with 1 ml of *Fol* bud cell (microconidia) suspension (10^7^ cells/ml) in 50 ml tubes. The tubes were vortexed vigorously and the soil were air-dried in a petri dish at room temperature overnight.

### DNA isolation

DNA isolation from fungal mycelium was performed as described before^35^. Briefly, fungal mycelia were ground in liquid nitrogen and mixed with 5 ml of DNA extraction buffer (0.7 M NaCl, 10 mM EDTA, 1% [w/v] SDS, 50 mM Tris–HCl pH 7.5). After vortexing for 2 min, 2 ml of PCI (phenol/chloroform/isoamyl alcohol, 25:24:1, v/v) was added and mixed by inverting the tube for 2 min. After centrifuging at 2,470 × g for 10 min, supernatant was mixed with an equal volume of PCI and re-centrifuged. Supernatant were mixed with an equal volume of chloroform and followed by centrifugation 2,470 × g, and then DNAs in the supernatant were precipitated in 99.5% ethanol with 0.3 M sodium acetate (pH 5.0), and centrifuged at 2,470 g to harvest the DNAs. DNA pellet was washed by 1 ml of 70% ethanol. DNA was dissolved with distilled water and stored at −30 °C before use. DNA concentration was determined by Nanodrop ND-1000 spectrophotometer (Thermo Scientific, Waltham, MA).

For DNA isolation from soil, Fast-DNA® Spin kit for soil (Bio101, Vista, CA, USA) with skim milk was used according to the manufacturer’s instructions and the methods described in the previous report^36, 37^.

### Design of a LAMP primer set and joint DNAs

A primer set for LAMP reaction were designed based on the *SIX3* (GenBank accession number: AM234063.1) gene sequence of *Fol* using the PrimerExplorer V4 software (http://primerexplorer.jp). The primer set comprised two outer primers (SIX3-F3 and SIX3-B3), two inner primers (SIX3-FIP and SIX3-BIP), and two loop primers (LF and LB). Nucleotide sequence of all primers were presented in Table 1. Two joint DNAs, Joint DNA 1 and Joint DNA 2, whose 3′-end binds to UQprobe-G, were designed for binding to two partial regions of *SIX3* sequences, nts. 113–129 or 127–149, respectively, with respect to the first nucleotide of its translation initiation codon. Positions of all primers were shown in Fig. 1. All primers and joint DNA were synthesized by Sigma-Aldrich (Tokyo, Japan). Nucleotide sequences of UQprobe-G is protected by Patent No. US7427672 B2, EP1661905 A1 and JP4731324.

### LAMP assay

LAMP reaction with the universal QProbe system was carried out with the previously described methods with slight modifications^38^. Briefly, the LAMP reaction mixture (25 µl) contained 1.4 mM each dNTP, 20 mM Tris–HCl (pH 8.8), 10 mM KCl, 8 mM MgSO_4_, 10 mM (NH_4_) _2_SO_4_, 0.1% Tween 20, and 0.8 M betaine, 0.2 µM each of F3 and B3 primers, 1.6 µM each of FIP and BIP primers, 0.8 µM each of Loop F and Loop B primers, 0.14 µM of UQprobe-G (J-Bio21, Tokyo, Japan), 0.4 µM of Joint DNA 1 or 2, *Bst* DNA polymerase (8 U) (Nippon Gene, Tokyo, Japan) and DNA (30 ng) as a template. LAMP reaction at 66 °C, as well as a subsequent melting curve analysis from 30 or 35 °C to 95 °C with a decrement of 0.2 °C per second, were performed by Genie II (OptiGene, Horsham, UK). Conventional LAMP reactions with *SIX4* primer set were conducted as described in a previously report^18^, with the Fluorescent detection reagent (Eiken Kagaku, Tokyo, Japan) as a fluorescent dye instead of UQprobe-G. Conventional LAMP reaction with *SIX3* primer set mixture, which contains above-mentioned except UQprobe-G and joint DNAs was incubated at 59–66 °C for 60 min followed by 10 min of incubation at 98 °C with Genie II (OptiGene) to detect and monitor fluorescence.

### PCR reaction

PCR reaction was performed in a reaction volume (10 µl) containing 1.25 units of Ex *Taq* polymerase (TaKaRa, Shiga, Japan), 0.5 µM each primer, 1 × Ex *Taq* buffer, 0.2 mM each dNTP and fungal genomic DNA (30 ng) using TaKaRa PCR Thermal Cycler Dice (TaKaRa). PCR conditions with *SIX3* LAMP outer primers was as follows: initial denature at 94 °C for 2 min, 30 cycle of denaturation at 98 °C for 30 s, annealing at 59 °C for 30 s and extension at 72 °C for 1 min, and a final extension at 72 °C for 6 min. PCR with the *SIX4* primer set was conducted as described in the previous report^11^. PCR products were electrophoresed on a 1% agarose gel, stained with ethidium bromide and visualized under UV irradiation.

### Plasmid construct

To generate *SIX3* gene containing a single point mutation (G137C), two PCR fragments harboring the mutation were amplified by using *Fol* race 1 gDNA (30 ng) and primers SIX3 F, SIX3 R, G137CF and G137CR, which were designed for introducing the mutation in the PCR product (Table 1). PCR condition was: initial denaturation at 94 °C for 2 min, followed by 30 cycles of denaturation at 98 °C for 10 s, annealing at 60.7 °C for 30 s and extension at 72 °C for 1 min, and final extension at 72 °C for 7 min. PCR products were purified by Wizard SV Gel and PCR Clean-Up System (Promega, Madison, WI, USA). The purified PCR products (3 ng) were ligated by recombinant PCR^39^. Constitution of PCR mixture was the same as above. The PCR conditions consisted of an initial denaturation at 94 °C for 2 min, annealing at 98 °C for 10 s and extension at 63 °C for 30 s and final extension at 72 °C for 1 min and 72 °C for 7 min. PCR product was cloned into pGEM-T easy vector (Promega), which was subsequently transformed into *Escherichia coli* Hit DH5α (RBC Bioscience, Taipei, Taiwan). Resultant transformants were selected by colony PCR with SIX3 F and SIX3 R primers followed by plasmid DNA extraction from the isolates with Wizard Plus SV PureYield Minipreps DNA Pulification System (Promega). Sequencing to confirm the mutation was performed with BigDye terminator v3.1 cycle sequencing system kit (Applied Biosystems, Foster City, CA, USA) as followed by previous report^40^ and 3130 × 1 Genetic Analyzer (Applied Biosystems).

## Electronic supplementary material

Supplementary Figure 1
