# Supplementary material for: Novel loop-mediated isothermal amplification (LAMP) assay with a universal QProbe can detect SNPs determining races in plant pathogenic fungi
Source: Sci Rep. 2017 Jun 26;7:4253. doi: 10.1038/s41598-017-04084-y (PMC5484703; doi:10.1038/s41598-017-04084-y)
Supplement: Supplementary file 1 — Supplementary Figure 1 [file 41598_2017_4084_MOESM1_ESM.pdf]

Supplementary information

Novel loop-mediated isothermal amplification (LAMP) assay with a universal QProbe can detect SNPs determining races in plant pathogenic fungi

Yu Ayukawa, Saeri Hanyuda, Naoko Fujita, Ken Komatsu,Tsutomu Arie

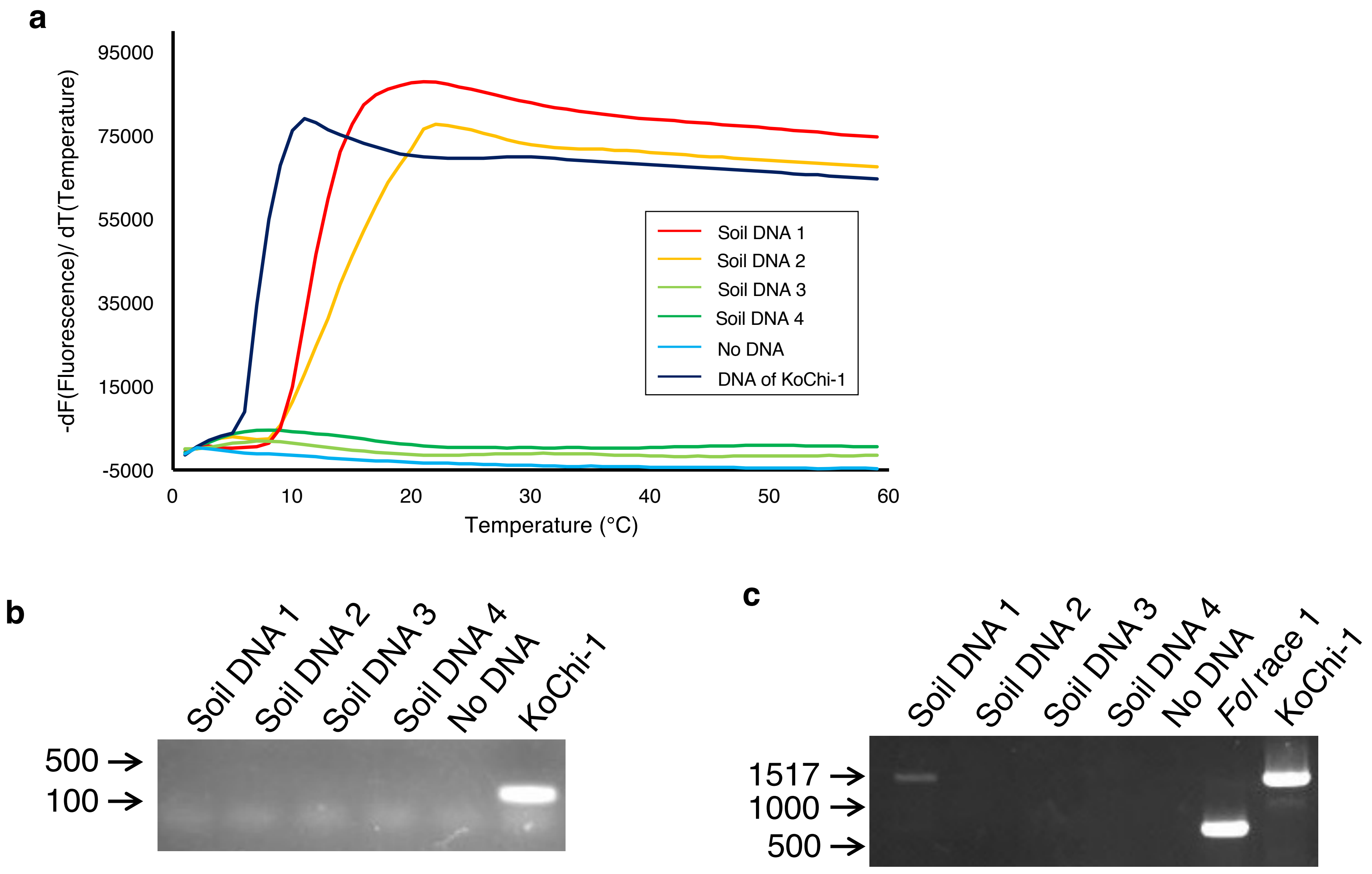

Supplementary Figure S1.
Comparison of conventional LAMP (a) and PCR to detect *SIX3* (b) and *SIX4* (c) from field soil DNA
(a) Conventional LAMP with a *SIX4* primer set. (b and c) Agarose gel electrophoresis of PCR products amplified with *SIX3* primers (b) and *SIX4* primers (c).
